# Optimal classifier selection and negative bias in error rate estimation: an empirical study on high-dimensional prediction

**DOI:** 10.1186/1471-2288-9-85

**Published:** 2009-12-21

**Authors:** Anne-Laure Boulesteix, Carolin Strobl

**Affiliations:** 1 Department of Statistics, University of Munich, Ludwigstr 33, D-80539 Munich, Germany; 2 Sylvia Lawry Centre for Multiple Sclerosis Research, Hohenlindenerstr 1, D-81677 Munich, Germany; 3 Department of Medical Informatics, Biometry and Epidemiology, University of Munich, Marchioninistr 15, D-81377 Munich, Germany

## Abstract

**Background:**

In biometric practice, researchers often apply a large number of different methods in a "trial-and-error" strategy to get as much as possible out of their data and, due to publication pressure or pressure from the consulting customer, present only the most favorable results. This strategy may induce a substantial optimistic bias in prediction error estimation, which is quantitatively assessed in the present manuscript. The focus of our work is on class prediction based on high-dimensional data (e.g. microarray data), since such analyses are particularly exposed to this kind of bias.

**Methods:**

In our study we consider a total of 124 variants of classifiers (possibly including variable selection or tuning steps) within a cross-validation evaluation scheme. The classifiers are applied to original and modified real microarray data sets, some of which are obtained by randomly permuting the class labels to mimic non-informative predictors while preserving their correlation structure.

**Results:**

We assess the minimal misclassification rate over the different variants of classifiers in order to quantify the bias arising when the optimal classifier is selected a posteriori in a data-driven manner. The bias resulting from the parameter tuning (including gene selection parameters as a special case) and the bias resulting from the choice of the classification method are examined both separately and jointly.

**Conclusions:**

The median minimal error rate over the investigated classifiers was as low as 31% and 41% based on *permuted uninformative *predictors from studies on colon cancer and prostate cancer, respectively. We conclude that the strategy to present only the optimal result is not acceptable because it yields a substantial bias in error rate estimation, and suggest alternative approaches for properly reporting classification accuracy.

## Background

It is well-known that almost all published studies present positive research results, as outlined by Kyzas et al [1] for the special case of prostate cancer. In the case of microarray studies, that often focus on the identification of differentially expressed genes or the construction of outcome prediction rules, this means that almost all studies report at least a few significant differentially expressed genes or a small prediction error, respectively.

According to Ioannidis [2], " [...] most published research findings are wrong". This may be partly due to the editorial policy of many journals which accept almost only papers presenting positive research results (except perhaps recent initiatives like the *Journal of Negative Research Results in Medicine*). Authors are thus virtually urged to "find something significant" in their data, which encourages the publication of wrong research findings due to a variety of technical and statistical pitfalls. Microarray studies are especially subject to such mechanisms and known to yield "noise discovery" [3].

Technical challenges that particularly affect microarray studies include, e.g. technical errors in the lab, problems with image analysis and normalization. Statistical pitfalls and biases of studies on microarray-based prediction are equally diverse. A problem well covered in the literature is the "small *n *large *p*" dimensionality problem (also referred to as "*n *≪ *p*", i.e. less observations than variables). In univariate analyses for identifying differentially expressed genes, the multiple testing problem resulting from high dimensionality can be addressed, e.g. by means of approaches based on the false discovery rate [4,5]. In the context of microarray-based prediction, another important statistical pitfall is incomplete cross-validation (CV), as pointed out by numerous authors [6-10]: if the selection of relevant variables is performed before cross-validation using all available observations, the cross-validated error rate is quite naturally optimistically biased. Most recent studies take this important point into account, either by performing variable selection for each CV iteration successively or by using class prediction methods involving an intrinsic variable selection step, like the Lasso [11]. Hence, we do not address again the problem of incomplete CV in the present article.

The reported classification error rate can also be lowered artificially by selecting the values of tuning parameters a posteriori, i.e. on the basis of the computed CV error rates. Doing this, one selects the "best" version of a classifier and evaluates it using the same data, which of course leads to an underestimation of the error rate (named "bias source I" in our present paper). A quantitative study on this topic can be found in [12]. Note that the problem of optimal parameter selection affects not only microarray research but also classical medical studies based on conventional low-dimensional predictors, although probably not as dramatically. A particular parameter that is especially crucial in the analysis of high-dimensional data is the number of selected variables (if variable selection is performed). In many studies, it is chosen a posteriori based on the CV results, thus inducing biases in the reported error rate. Another source of bias (named "bias source II" in our paper) that is related to, but more global than optimal parameter selection, is the optimal selection of the classification method itself from the wide range of classifiers that are available for the analysis of microarray data today (e.g. support vector machines, random forest or *L*_2 _penalized logistic regression). This again is an issue that, in principle, can be encountered in all types of medical studies, but affects microarray studies more drastically. Whereas standard statistical approaches - for instance logistic regression for class prediction problems - have become the methodological "gold standard" in conventional medical statistics and allow a comparatively fair evaluation of research results, the field of microarray data analysis is characterized by the lack of benchmark standard procedures and a huge and heterogeneous amount of methods - ranging from adaptations of standard statistical procedures to computer intensive approaches adopted from machine learning - whose respective merits and pitfalls remain partly unexplored. This is particularly true for studies involving class prediction problems, i.e. when the goal is to derive a classification rule for predicting the class membership (typically the disease outcome) of patients based on their microarray transcriptome data.

In this context, if the sample is not large enough to put aside a validation data set, it is common practice to evaluate the performance of classifiers based on techniques like cross-validation (CV) including leave-one-out (LOO) CV as a special case, repeated splitting into learning and test data sets, or bootstrap sampling. See the methods section for more details on the cross-validation technique used here and [13] for an extensive review on cross-validation and resampling techniques in general. However, it is not sufficient to use correct methods together with a correct internal CV scheme. Evaluating several classification methods in cross-validation and then reporting only the CV results obtained with the classifier yielding the smallest error rate is an incorrect approach [14], because it induces an optimistic bias. In their dos and donts list, Dupuy and Simon [14] recommend to "report the estimates for all the classification algorithms if several have been tested, not just the most accurate." They discourage from optimizing the choice of the classification algorithm based on the obtained results.

In this article, we empirically investigate the consequences of such an optimization. We report the results of an experiment that allows us to quantify the optimistic bias induced by optimal tuning parameter selection (bias source I) and optimal selection of the classification method (bias source II) in a realistic setting based on original microarray data. After we have illustrated the drastic effect of optimal classifier selection, we discuss alternative ways to report results of class prediction studies when no validation set is available, and give suggestions for good scientific practice in this context.

In our experiment we compute the misclassification rate of a total of 10 usual classification methods (k-nearest-neighbors, linear discriminant analysis, Fisher's discriminant analysis, diagonal linear discriminant analysis, partial least squares followed by linear discriminant analysis, neural networks, random forests, support vector machines, shrunken centroid discriminant analysis and *L*_2_-penalized regression) based on cross-validation. Some of these 10 classification algorithms are combined with preliminary variable selection or/and used with different plausible tuning parameter values successively. The aim is to investigate the different sources of biases resulting from optimal selection (optimal choice of tuning parameters including gene selection and optimal choice of the classification method) and their relative importance. All the considered procedures are classical approaches, most of which have already been used in published medical studies. For the sake of reproducibility, all our analyses are based on the freely available Bioconductor package CMA version 0.8.5 [15] which is described extensively in [16].

The classifiers are applied to original and modified data sets, some of which are obtained by permuting the class labels of real microarray data sets. We then assess the minimal misclassification rate over the results of the different variants of classifiers in order to quantify the bias arising when the optimal classification method and/or its tuning parameters are selected a posteriori in a data-driven manner. The permutation of the class labels is used to mimic data sets under the global null hypothesis that none of the genes are differentially expressed with respect to the response class. This approach thus provides non-informative microarray data that, however, preserve their realistic correlation structure, and can serve as a "baseline" to quantify the bias induced by optimal classifier selection.

## Methods

### Generating the data

Three types of data sets are used in the present study: i) non-modified real data sets, ii) data sets with permuted class labels, to mimic non-informative microarray data with a realistic correlation structure, and iii) subsamples of non-modified real data sets including 60, 65, 70, 75, 80, 85, 90 or 95% of the original sample, to be able to evaluate potential effects of the sample size. To average out the random fluctuations due to the permutation process, we consider *niter *= 20 balanced permutations successively. The term "balanced" permutation here means that within each true class, a fraction of about *N*_1_/*N *of the observations are randomly assigned to class 1, and a fraction of *N*_0_/*N *of the observations to class 0, where *N*_0 _and *N*_1 _denote the numbers of observations from classes 0 and 1 respectively in the entire sample. By doing so, we make sure that none of the permuted class label vectors is similar to the true class label vector by chance.

Two real-life data sets are considered successively: the well-known benchmark colon data set [17], and the prostate cancer data set [18].

### Included classifiers

This study includes only well-known classifiers that are widely used in the context of class prediction in medical studies and yield acceptable accuracies in "neutral comparison studies" (see [13] for criteria defining such studies). For instance, methods like quadratic discriminant analysis are excluded because they seem to perform poorly [19] and are not often used in practice.

We consider a total of 124 classifiers. By classifier, we mean the combination of the variable selection procedure (if any) and the method used to construct the prediction rule, potentially including different tuning parameter settings. More specifically, we use the ten classification methods outlined below:

• **KNN **(*k*-nearest-neighbors): the standard nearest-neighbor approach [20]. We consider successively *k *= 1, 3, 5, which are all usual choices when the method is used on small sample data.

• **LDA **(Linear Discriminant Analysis): standard linear discriminant analysis with normality assumption and common within-group covariance matrix, as summarized in [19,20].

• **FDA **(Fisher's Discriminant Analysis): standard Fisher's discriminant analysis [19].

• **DLDA **(Diagonal Linear Discriminant Analysis): the same as LDA, except that the common within-group covariance matrix is assumed to be diagonal. The feature makes it applicable to data with *n *≪ *p*.

• **PLSLDA**: Partial Least Squares (PLS) dimension reduction (see [21] for an overview) followed by linear discriminant analysis using the PLS components as predictors [22]. We set the number of PLS components to *ncomp *= 2 and *ncomp *= 3 successively.

• **NNET**: neural networks with one hidden layer.

• **RF **(Random Forests): the random forest method [23] aggregating a large number of classification trees obtained from different subsamples and with random selection of candidate predictors at each split. The number of trees is set to *ntree *= 1000, whereas the number *mtry *of candidate predictors considered at each split is set either to *mtry *=  (default setting for classification), ,  or , which are all sensible values [24].

• **SVM **(Support Vector Machines): the well-known kernel method by Vapnik [25]. Tuning parameters include the kernel function (for instance linear and radial), the cost (for both linear and radial kernels) and the parameter controlling the width of the radial kernel. In this paper, we consider linear kernels only (the default in the R function svm from the e1071 package), which are known to perform well. The cost is considered as a tuning parameter and optimized via internal 3-fold-cross-validation.

• **PAM **(Shrunken Centroid Discriminant Analysis, also called "Prediction Analysis with Microarrays"): the nearest shrunken centroids method [26]. The shrinkage parameter is optimized via internal 3-fold-cross-validation.

• **L**_2_: the *L*_2_-penalized logistic regression approach (also called ridge regression). In contrast to Lasso, this approach does not yield sparse models. The penalty parameter is optimized via internal 3-fold-cross-validation.

The methods used to construct prediction rules are summarized in Table 1 together with the different numbers of genes and parameter values considered in this study. All the methods are well-known and reported to perform reasonably well in the literature. Note that we could also have included methods like the Lasso [11] and elastic net [27]. However, we do not consider them in the present study because they show convergence problems in some cases or/and are computationally intensive. Other methods which could have been included are tree-based and componentwise boosting [28], or alternative dimension reduction techniques (see [29] for a synthetic overview). We only include a limited number of methods to keep the scope of the study manageable, but also to mimic the scope of methods that an average biostatistician/bioinformatician would realistically be able to apply to his/her data in a limited time-frame. Some arbitrariness in the choice of the methods is unavoidable, but we feel that the current selection covers the spectrum of methods currently available and attractive for microarray studies reasonably well. In particular, it includes both purely statistical approaches and machine learning algorithms.

**Table 1:** Summary of the considered candidate classifiers

Method	Type	Number of genes p*	Function	Fixed parameters	Parameters tuned via CV
KNN	1	20, 50, 100, 200, 500	knnCMA	*k *= 1, 3, 5	

LDA	2	10, 20	ldaCMA		
FDA	2	10, 20	fdaCMA		

DLDA	3	20, 50, 100, 200, 500	dldaCMA		
PLSLDA	3	20, 50, 100, 200, 500	plsldaCMA	*ncomp *= 2, 3	
NNET	3	20, 50, 100, 200, 500	nnetCMA		

RF	4		rfCMA	*mtry *= ,, ,	
linear SVM	4		svmCMA		cost
PAM	4		pamCMA		shrinkage parameter
*L*_2_	4		plrCMA		penalty

Column 1: Acronym of the method. Column 2: Type of the method regarding preliminary variable selection. Column 3: Number of selected genes p* (if preliminary variable selection is performed). Column 4: Name of the function in the CMA package. Column 5: Name and values of the fixed parameters. Column 6: Name of the parameters tuned using internal 3-fold-cross-validation.

### Tuning parameters

One of the two sources of bias investigated in this paper is the optimal selection of tuning parameters including as a special case the number of selected genes and the gene selection method - if preliminary variable selection is performed.

#### Preliminary variable selection

Regarding variable selection, classification methods can be divided into several categories:

1. Methods that do not take each variable's prediction strength into account, such as *k*-nearest-neighbors. Such methods almost always yield poor prediction accuracies when applied to noisy data. For instance, in the nearest neighbors approach, array-to-array distances on which prediction is based are computed using all genes irrespectively of their discrimination power. Such methods should be combined with variable selection.

2. Methods that take the variables' prediction strength into account, but can technically not be applied to data with *n *≪ *p*, like linear discriminant analysis. For such methods to be applicable, previous variable selection is necessary and the number of selected variables should be smaller than the number of observations.

3. Methods that take the variables' prediction strength into account and can technically be applied to data with *n *≪ *p*, but usually perform better on a reduced subset of relevant genes, like diagonal linear discriminant analysis (DLDA). By definition, DLDA gives more weight to genes with a high signal-to-noise ratio. However, genes with poor or no discriminating power behave as noise and usually decrease classification accuracy substantially.

4. Methods that take the variables' prediction strength into account, can technically be applied to data with *n *≪ *p *and whose prediction accuracy is usually not improved by preliminary variable selection. Such methods include, e.g. nearest shrunken centroids or the Lasso, that perform variable selection intrinsically. Note, however, that preliminary variable selection may be necessary in some cases in practice for computational reasons, especially when the number of genes reaches several tens of thousands as common in modern data sets.

In the present study, we consider only smaller data sets with *p *< 10, 000 genes, which makes preliminary variable selection for the retained methods of type 4 unnecessary from a computational point of view. For methods of types 1 to 3, previous variable selection is applied with different (round) numbers of genes p*: 10 and 20 genes when the method requires *n *> p* and 20, 50, 100, 200, or 500 genes when the method can cope with *n *<p*, see Table 1 for an overview. In our study, three very common selection criteria are used successively: the absolute value of the two-sample t-statistic, the absolute value of the limma statistic [30] and the absolute value of the normalized two-sample Wilcoxon statistic. Note that further methods could have been considered, such as the "traditional" Golub criterion or more sophisticated multivariate approaches, e.g. based on random forests [24]. In our experiment, however, we focus on the most standard approaches, because we consider it realistic that a statistician, who wants to try a large number of procedures, would prefer those that are freely available or easy to implement, computationally efficient and conceptually simple.

#### Other tuning parameters

Apart from preliminary variable selection, some of the considered classification methods involve tuning parameters, for instance the shrinkage parameter of the PAM method, the penalty in *L*_2 _penalized regression, and the cost parameter in linear SVM. In our study, these method parameters are tuned by performing an internal 3-fold-cross-validation using the learning set only, because i) it is a commonly recommended strategy [31], and ii) there is no reliable "gold standard" for these parameters.

We also consider classification methods involving tuning parameters for which default values are expected to work reasonably well with most data sets. In this case, parameter tuning through inner cross-validation is not as essential and well-established as for methods like penalized regression. In our study, these methods include KNN (with the number *k *of neighbors as parameter), random forests (with the number *mtry *of candidate splitting variables considered at each split as parameter), and PLSLDA (with the number *ncomp *of PLS components as parameter). For these three methods, we consider a few standard values of the tuning parameter successively and investigate the bias resulting from optimal selection, see Table 1 for an overview. Some subjectivity and arbitrariness in the study design is unavoidable, but this setup can give a realistic example of the bias that is induced when certain choices in model selection are based on optimizing performance on the learning data.

#### The 124 variants of classifiers

On the whole, we obtain 124 variants of classifiers from the combination of 10 classification methods, some in combination with different numbers p* of preselected genes, each selected with one of three selection criteria (t, limma or Wilcoxon statistic), and different tuning parameter settings. The number 124 is obtained as the sum for the 10 considered methods of

where *G *is the number of values of p* (i.e. the number of values in the third column of Table 1 with the convention *G *= 0 when no gene selection is performed), three is the number of considered gene selection methods (t-statistic, Wilcoxon, limma), and *T *is the number of considered values for the fixed tuning parameters (i.e. the number of values in the fifth column of Table 1 with the convention *T *= 0 if there are no such fixed parameters). This formula, yielding a total of 124, is given explicitly in Table 2.

**Table 2:** Formula yielding a total of 124 classifiers

KNN:	5 values of p*	×	3 gene selection criteria	×	3 values of *k*	+
LDA:	2 values of p*	×	3 gene selection criteria			+
FDA:	2 values of p*	×	3 gene selection criteria			+
DLDA	5 values of p*	×	3 gene selection criteria			+
PLSLDA:	5 values of p*	×	3 gene selection criteria	×	2 values of *ncomp*	+
NNET:	5 values of p*	×	3 gene selection criteria			+
RF				×	4 values of *mtry*	+
SVM						+
PAM						+
*L*_2_						=
						124 classifiers

This table explains how we obtain a total of 124 classifiers.

### Cross-validation (CV)

It is well-known that the prediction accuracy of a classifier should not be evaluated based on the data that were used for its construction. Instead, if no independent validation set is available, the classifier should be evaluated through a CV-like technique considering several pairs of learning and test sets successively. In the present experiment, we use one of the most widely used evaluation schemes: *k*-fold cross-validation. In *k*-fold cross-validation (for instance *k *= 5), the available data set is split in *k *approximately equally sized subsets. At each of the *k *iterations, one of these subsets is considered as a test data set, while the union of the *k *- 1 other subsets forms the learning set. The cross-validated error rate is then computed by averaging the error rates obtained in all *k *iterations. The reader is referred to [13,32] for more technical details and critical discussions of cross-validation or related evaluation procedures. In the present study, the number *k *of iterations is set to the standard value *k *= 5.

### Implementation

In this study, we use the Bioconductor package 'CMA' [15] described in [16]. Our R codes are publicly available at http://www.ibe.med.uni-muenchen.de/organisation/mitarbeiter/020_professuren/boulesteix/errorratebias.

## Results and Discussion

### Data sets and real data analysis results

We first analyze the well-known colon cancer microarray data set [17] including *p *= 2000 genes for 22 normal and 40 tumor tissues (*n *= 62) that is available from the Bioconductor package 'colonCA'. This data set was analyzed in numerous classification-based articles including comparison studies [33]. The obtained error rates usually range between 10% and 20%. Note that the results from different studies are difficult to compare, since they are all based on different evaluation designs (for instance CV, LOOCV, bootstrap, etc.) and different variable selection approaches.

We apply the 124 classifiers to this data set and obtain error rates ranging from 11% to *> *35%, see Table 3 (top) for the results obtained without preliminary variable selection (for RF, SVM, PAM, *L*_2_) or with variable selection based on the t-statistic (for KNN, LDA, FDA, DLDA, PLSLDA, NNET). The results with the Wilcoxon statistic and the limma statistic are similar. As can be seen from Table 3, the different methods yield noticeably different results.

**Table 3:** Results of the real data study, colon data

Colon		p*	10	20	50	100	200	500	2000
Method	Parameter								
KNN	*k *= 1		-	0.19	0.21	0.24	0.18	0.23	-
KNN	*k *= 3		-	0.18	0.15	0.16	0.16	0.16	-
KNN	*k *= 5		-	0.18	0.16	0.19	0.15	0.13	-

LDA			0.19	0.21	-	-	-	-	-

FDA			0.18	0.21	-	-	-	-	-

DLDA			-	0.15	0.16	0.13	0.18	0.24	-

PLSLDA	*ncomp *= 2		-	0.16	0.16	0.16	0.18	0.16	-
	*ncomp *= 3		-	0.18	0.16	0.13	0.16	0.18	-

NNET			-	0.35	0.35	0.34	0.37	0.34	-

RF	*mtry *=		-	-	-	-	-	-	0.18
	*mtry *=		-	-	-	-	-	-	0.18
	*mtry *=		-	-	-	-	-	-	0.18
	*mtry *=		-	-	-	-	-	-	0.18

SVM			-	-	-	-	-	-	0.13

PAM			-	-	-	-	-	-	0.11

*L*_2_			-	-	-	-	-	-	0.18

**Prostate**		p*	**10**	**20**	**50**	**100**	**200**	**500**	**5908**
Method	Parameter								

KNN	*k *= 1		-	0.12	0.15	0.14	0.10	0.12	-
KNN	*k *= 3		-	0.08	0.08	0.07	0.07	0.09	-
KNN	*k *= 5		-	0.07	0.09	0.08	0.09	0.10	-

LDA			0.08	0.08	-	-	-	-	-

FDA			0.08	0.08	-	-	-	-	-

DLDA			-	0.10	0.13	0.13	0.19	0.24	-

PLSLDA	*ncomp *= 2		-	0.06	0.08	0.06	0.08	0.08	-
	*ncomp *= 3		-	0.08	0.06	0.06	0.08	0.07	-

NNET			-	0.10	0.12	0.10	0.15	0.20	-
RF	*mtry *=		-	-	-	-	-	-	0.08
	*mtry *=		-	-	-	-	-	-	0.08
	*mtry *=		-	-	-	-	-	-	0.08
	*mtry *=		-	-	-	-	-	-	0.08

SVM			-	-	-	-	-	-	0.10

PAM			-	-	-	-	-	-	0.21

*L*_2_			-	-	-	-	-	-	0.09

CV error rates obtained with different methods for the colon data set (top) [17] and the prostate data (bottom) [18], with variable selection (if any) based on the t-statistic.

The second data set investigated in the present study is introduced by Singh et al [18] and includes 5908 genes for 50 normal and 52 prostate cancer tissues. We use the data preparation procedure described in [22]. This data set usually yields better accuracy than the colon data set, which may be partly due to the larger sample size, see for instance [22]. In the present study, the estimated error rates strongly vary across the classifiers. Whereas some well-known classifiers such as PAM yield poor accuracy (error rates of approximately 20%), others, such as partial least squares, yield error rates of approximately 6%, see Table 3 (bottom) for a summary.

Note that the relative performance of the different methods is substantially different in the two data sets. This is consistent with the widely acknowledged fact that there is no unique "best" classification method yielding top-performance for all data sets. Hence, it would be unwise to try only one classification method: the multiplicity issue investigated in this study is relevant.

### Permutation-based analysis

In this part of the analysis, we simulate non-informative gene expression data sets by permuting the class labels of the two data sets described above, thus mimicking non-informative microarray data with a realistic correlation structure. To average out variations due to the random permutation, 20 balanced permutations are considered successively. The two sources of bias (optimal selection of the tuning parameters and optimal selection of the classification method) are assessed separately in the two next subsections.

#### Bias source I: tuning parameters and gene selection

The aim of this part of the analysis is to assess the bias induced by optimally selecting the number of genes p*, the gene selection procedure and/or the tuning parameters based on the CV-results. For each of the 10 classification methods and each of the 20 permuted data sets, we consider the six following error rates:

**Approach A: **Minimal error rate over the different tuning parameter values (*k *= 1, 3, 5 for KNN, *ncomp *= 2, 3 for PLSLDA, *mtry *= , , ,  for RF), different numbers of genes and different gene selection methods.

**Approach B: **Minimal error rate over the different numbers of genes and different gene selection methods. In contrast to A, the tuning parameter is fixed for all methods involving a tuning parameter other than the number of genes (*mtry *= , , ,  for RF, *ncomp *= 2 for PLSLDA, and *k *= 5 for KNN).

**Approach C: **Minimal error rate over the different tuning parameter values (*k *= 1, 3, 5 for KNN, *ncomp *= 2, 3 for PLSLDA, *mtry *= , , ,  for RF) and different gene selection methods. In contrast to A, the number of genes is fixed (p* = 20 for the methods requiring *n *> *p*, p* = 100 for the other).

**Approach D: **Minimal error rate over the different tuning parameter values (*k *= 1, 3, 5 for KNN, *ncomp *= 2, 3 for PLSLDA, *mtry *= , , ,  for RF) and different numbers of genes. In contrast to A, the gene selection method is fixed (gene selection based on the t-statistic for all methods involving gene selection).

**Approach E: **Minimal error rate over the different tuning parameter values (*k *= 1, 3, 5 for KNN, *ncomp *= 2, 3 for PLSLDA, *mtry *= , , ,  for RF). In contrast to A, the number of genes and the gene selection method are fixed (gene selection based on the t-statistic, p* = 20 for the methods requiring *n *> *p*, p* = 100 for the other).

These minimal error rates are computed for all 20 permutation runs and for each of the 10 classification methods. For each classification method and each approach, the median over the 20 runs is given in Table 4. For comparison, Table 4 also gives the median error rate calculated from all 124 × 20 error rates obtained with the 124 classifiers for the 20 permutation runs (last column: F). The difference between this baseline and the minimal error rates A, B, C, D, and E can be interpreted as the bias induced by optimal selection. It can be seen from Table 4 that, for a given classification method, the optimization of the gene selection method, of the number of genes and of the tuning parameters contribute approximately equally to the bias: there is no unique source of bias. Let us consider the KNN method applied on the prostate data for illustration. The median minimal error rate over all parameter values (*k *= 1, 3, 5), all numbers of genes (p* = 20, 50, 100, 200, 500) and the three gene selection methods (t-statistic, Wilcoxon, limma) is 0.43. It equals 0.45 if one optimizes over the results obtained with *k *= 5 only (Approach B) and if one optimizes over the results obtained with p* = 100 only (Approach C). Optimization over the results obtained with the t-statistic as fixed gene selection method (Approach D) yields a median error rate of 0.47, while the optimization over the three tuning parameter values yields the higher value 0.50. Most importantly, if all 10 classification methods are considered simultaneously, the bias due to the optimization of the classification method dominates all the other sources of bias, as investigated in more details in the next section.

**Table 4:** Results of the permutation study

Colon	A	B	C	D	E	F
KNN	0.33	0.36	0.37	0.38	0.41	0.45

LDA	0.40	-	0.43	0.43	-	0.46

FDA	0.42	-	0.44	0.47	-	0.48

DLDA	0.36	-	0.41	0.42	-	0.44

PLSLDA	0.34	0.35	0.37	0.37	0.42	0.43

NNET	0.34	-	0.35	0.35	-	0.36

RF	0.40	0.40	-	-	-	0.42

SVM	0.37	-	-	-	-	0.37

PAM	0.36	-	-	-	-	0.36

*L*_2_	0.44	-	-	-	-	0.44

**All**	0.31	0.32	0.33	0.33	0.34	0.43


**Prostate**	**A**	**B**	**C**	**D**	**E**	**Baseline**

KNN	0.43	0.45	0.45	0.47	0.50	0.52

LDA	0.46	-	0.47	0.50	-	0.51

FDA	0.45	-	0.47	0.49	-	0.49

DLDA	0.46	-	0.49	0.49	-	0.51

PLSLDA	0.44	0.46	0.47	0.49	0.51	0.52

NNET	0.46	-	0.49	0.47	-	0.52

RF	0.52	0.54	-	-	-	0.54

SVM	0.57	-	-	-	-	0.57

PAM	0.54	-	-	-	-	0.54

^*L*^2	0.52	-	-	-	-	0.52

**All**	0.41	0.42	0.43	0.44	0.46	0.52

Colon data set [17] and prostate data set [18], with variable selection (if any) based on the t-statistic. **Approach A: **Minimal error rate over the different tuning parameter values (*k *= 1, 3, 5 for KNN, *ncomp *= 2, 3 for PLSLDA, *mtry *= , , ,  for RF), different numbers of genes and different gene selection methods (median over the 20 runs). **Approach B: **Minimal error rate over the different numbers of genes and different gene selection methods (median over the 20 runs). **Approach C: **Minimal error rate over the different tuning parameter values (*k *= 1, 3, 5 for KNN, *ncomp *= 2, 3 for PLSLDA, *mtry *= , , ,  for RF) and different gene selection methods (median over the 20 runs). **Approach D: **Minimal error rate over the different tuning parameter values (*k *= 1, 3, 5 for KNN, *ncomp *= 2, 3 for PLSLDA, *mtry *= , , ,  for RF) and different numbers of genes (median over the 20 runs). **Approach E: **Minimal error rate over the different tuning parameter values (*k *= 1, 3, 5 for KNN, *ncomp *= 2, 3 for PLSLDA, *mtry *= , , ,  for RF) (median over the 20 runs). **Approach F: **Median of all 124 × 20 calculated error rates.

#### Bias source II: choice of the classification method

In this subsection, we consider the second source of bias, namely the optimal choice of the classification method. Here the tuning parameters are either optimized through a correct inner cross-validation procedure or fixed to a single plausible value in order to handle all 10 classifiers equally. The minimal error rate is derived over the resulting 10 error rates. The 10 considered combinations are as follows: KNN with *k *= 5 and p* = 100 genes selected based on the t-statistic, LDA with p* = 20 genes selected based on the t-statistic, FDA with p* = 20 genes selected based on the t-statistic, DLDA with p* = 100 genes selected based on the t-statistic, PLSLDA with *ncomp *= 2 and p* = 100 genes selected based on the t-statistic, NNET with p* = 100 genes selected based on the t-statistic, RF with *mtry *= , SVM with cross-validated cost, PAM with cross-validated shrinkage parameter, *L*_2 _with cross-validated penalty. Note that p* = 20 genes were used for the methods of type 2 that require *n *> *p*.

For each of the 20 permutation runs, the minimal error rate  over these 10 classifiers is derived, where the exponent (*H*_0_) in  indicates that this error rate was computed based on permuted data, i.e. under the null-hypothesis of no association between the response class and the predictors.

Moreover, we also derive the minimal error rate over the 6 × 3 + 4 = 22 classifiers yielded as combinations of a classification method and a gene selection method (for the six classification methods KNN, LDA, FDA, DLDA, NNET, with fixed p* = 100). The rationale behind this is that it makes sense to consider the gene selection procedure as a component of the classification method rather than as a tuning parameter. This minimal error rate is denoted as .

**Figure 1:**
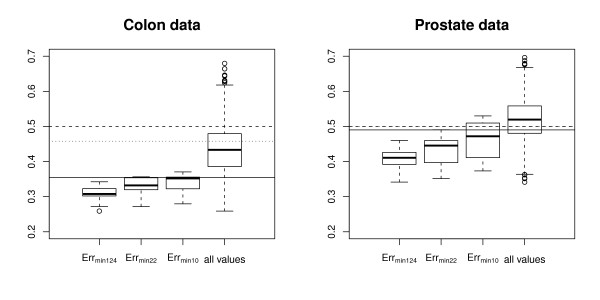
**Permutation-based analyses**. Alon's colon cancer data (left) and Singh's prostate cancer data (right). Boxplots of the minimal error rates ,  and  for the 20 permutations, and of all the error rates obtained with the 124 classifiers for the 20 permutations = 124 × 20 points (right). The three horizontal lines represent the three baseline error rates defined as follows: the error rate obtained by assigning all observations to the majoritary class (plain), the error rate obtained by randomly assigning *N*_0 _observations to class 0 and *N*_1 _observations to class 1 (dotted), and 50% (dashed). **Main conclusion: **The minimal error rate is much lower than all three baseline error rates, and a large part of this bias is due to the optimal selection of the classification method.

The distribution of  and  over the 20 permutation runs is displayed in form of boxplots in Figure 1 for the colon data set (left panel) and the prostate data set (right panel). For comparison, Figure 1 also shows the boxplot of the minimal error rate  obtained by minimizing over all 124 classifiers (left box) and the distribution of all 124 × 20 = 2480 error rates obtained by running the 124 classifiers on the 20 permuted data sets (right box).

Additionally, Figure 1 includes three theoretical baseline values corresponding to i) an error rate of 50%, ii) the mean error rate 2*N*_0_*N*_1_/*N*^2 ^which would be obtained by randomly assigning *N*_0 _observations to class *Y *= 0 and *N*_1 _observations to class *Y *= 1 (dotted line), and iii) the error rate min(*N*_0_, *N*_1_)/*N *which would be obtained by assigning all the observations to the majoritary class (dashed line).

As expected, Figure 1 shows that the global minimal error rate  over the 124 classifiers is affected by a strong bias compared to the three baselines. A perhaps more striking result is that  (obtained by minimizing over the 10 classification methods) and  (obtained by minimizing over the 22 combinations of classification methods and gene selection methods) also carry a substantial bias, especially in the colon data set. Unsurprisingly,  and  are greater than , because optimization is performed over 10 and 22 error rates, respectively, instead of 124. However, it is clear from Figure 1 that a large part of the total bias is due to the optimization of the classification method. If one additionally optimizes the tuning parameters (including the number of genes as a special case), the bias only increases moderately.

Note that the bias compared to the baseline 50% is higher for the colon data set than for the prostate data set, which can be at least partly explained by the difference of the sample sizes and the ratio of class frequencies (almost 1:1 in the prostate data set, but ≈2:1 for the colon data set). More precisely, under the null-hypothesis of non-informative gene expression data, the median minimal error rate is 31% for the colon data set, and 41% for the prostate data set.

### Outlook: subsample analysis

As an outlook, the bias over the 124 classifiers is also assessed based on subsamples drawn randomly from the original data set. We successively consider subsamples corresponding to 60, 65, 70, 75, 80, 85, 90 or 95% of the sample size *n*. For each proportion of the original sample size, the procedure is repeated 20 times to average out variability due to random subsampling. The minimal error rate over the 124 classifiers is derived for each subsample.

**Figure 2:**
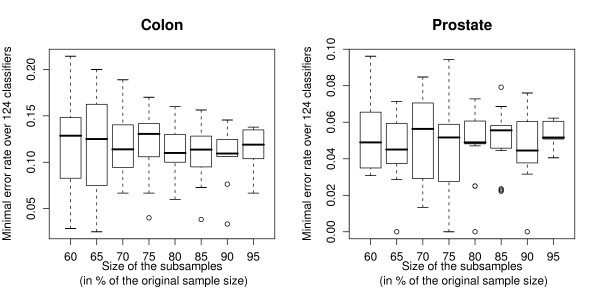
**Subsample analyses**. Alon's colon cancer data (left) and Singh's prostate cancer data (right). Boxplots of the minimal error rate over the 124 classifiers for each subsample size (each boxplot corresponds to 20 error rate estimates). **Main conclusion: **The median minimal error rate does not seem to increase with decreasing sample size.

Figure 2 represents the boxplots of the minimal error rate  over the 124 classifiers for each subsample size (each boxplot corresponds to 20 error rate estimates) for the colon cancer data [17] and the prostate cancer data [18]. It can be seen that the median minimal error rate is approximately 12-13% and 5-6% for all subsample sizes for the colon and the prostate data, respectively, i.e. does not increase with decreasing sample size as usually expected in prediction. This observation is the result of two competing effects. On the one hand, the error rate of each single classifier is inversely related to the sample size. On the other hand, when the sample size decreases, the variance of the 124 estimated error rates increases, thus decreasing the minimum over the 124 classifiers. On the whole, these two competing effects seem to approximately compensate, yielding a constant median minimal error rate for both data sets, as can be observed from Figure 2.

### Some solutions

From the results presented in the previous section, it is clear that one should definitely not report only the best result *Err*_*min*124_, because this strategy generates a considerable optimistic bias. In practice, the bias due to the optimal selection of the tuning parameters ("bias source I") is often addressed by nested cross-validation [12]. Within each cross-validation iteration, the best parameter value is determined based on an inner cross-validation procedure and the error rate is then computed for this parameter value. The final error rate estimate is obtained by averaging over the cross-validation iterations. Note that, doing that, one averages error rates obtained with different parameter values. As demonstrated by Varma and Simon [12], this approach correctly addresses "bias source I". Going one step further, one could theoretically consider the classification method as a (nominally scaled) tuning parameter and also address "bias source II" using nested cross-validation. However, besides substantial interpretation problems, this approach would be extremely computationally expensive and difficult to apply in practice for methods involving a tuning parameter that also has to be tuned via inner cross-validation. More research is needed before nested cross-validation can be recommended as a standard solution to bias source II. In order to avoid such biases in practical studies, we give the following recommendations, which partly overlap with those given in [14].

One should test the derived classifier on untouched validation data whenever possible. This approach consists of splitting the available data into i) a training set which is used to construct classifiers and select the "best" one and ii) a validation data, which is not opened until a final classifier has been selected based on the training data, in the vein of the validation strategy described by Daumer et al [34]. In practice, this is possible only when the sample is large enough, say, *n *≥ 150, a condition which is not fulfilled by all microarray studies. When a validation data set is available, the error rate estimates of the cross-validation procedure on the training set are of secondary importance. Cross-validation is only used for selecting the best classifier, i.e. for comparison purposes but not for estimating the error rate. The estimate of the error rate obtained from the validation data set is then unbiased, since this data set was not opened during the training phase. Note that the more classifiers are tried during the training phase, the more the validation accuracy will decrease compared to the best training accuracy, due to multiple comparisons effects.

If the study does not include any validation step with untouched data, one should not only report the results obtained with the best performing method/parameter combination/number of genes, as demonstrated in our study on original and modified real microarray data. In this situation, handling and reporting the results in a fair way is a complex task. Ideally, the considered classification methods should be documented in form of an analysis plan (in the vein of those written by pharmaceutical laboratories for their (pre-)clinical studies) before starting the data analysis. In this context, the publication of analysis plans on open access platform like *Nature Precedings *is to be encouraged [35]. However, analysis plans do not directly answer the question of how to report results from different classifiers. Reporting the results of all tried classifiers would be confusing, need too much space, and unavoidably raise the question of which estimate is the "right one". Reporting an average error rate would be a solution to these problems, but some arbitrariness remains in the choice of classifiers: by including variants of good or bad classifiers, one could almost arbitrarily decrease or increase the average error rate. Therefore the intuitive interpretability of an average error rate would require that the set of candidate classifiers is the same in all studies. Although some efforts have been made in the last few years to define a set of standard classifiers (see for instance the R packages 'MCRestimate' [36] and 'CMA' [15]), the standardization of candidate classifiers in medical research is still in its infancy.

Alternatively, we suggest an approach inspired from our permutation-based simulation design. Since it does not make much sense to focus on bad performing classifiers, we suggest to consider the minimal error rate over all tried classifiers as the main outcome of the study. However, to avoid hasty optimistic conclusions, we propose to compare this minimal error rate from the original data *Err*_*min *_with the median minimal error rate obtained from data sets with permuted class labels , as considered as a baseline in our simulation study. More precisely, we suggest to report the results of microarray studies in the following form: "By using microarray predictors, the minimum achievable prediction error for the colon data set could be reduced from 31% (baseline under *H*_0_) to 11%." This reporting scheme has two strong advantages: i) it automatically adjusts for the number of candidate classifiers and ii) it accounts for the fact that the error rate also depends on the class proportions.

## Conclusions

In this article, we have quantitatively assessed the bias induced by reporting only the error estimate of an optimally selected classifier. The focus here was on the classifier construction step, which is one of the most complex from a statistical point of view. However, the optimal choice of a method might take place at other levels of microarray preprocessing and analysis, too. Firstly, the normalization technique (e.g. MAS, RMA, etc for Affymetrix) can also be optimized, since there exist a number of normalization approaches which can be tested easily, for instance using standardized R packages from the Bioconductor platform. The choice of the normalization procedure may greatly affect the classification accuracy of prediction rules, perhaps even more than the choice of the classifier. However, each team probably has its preferred procedure and applies it systematically without switching to another one, even if the results are disappointing. Hence, the choice of the normalization technique is probably not an important source of optimal selection bias in the sense considered here.

Secondly, one could optimize the criterion used to filter out genes that are, e.g. not enough regulated. Several variants of selection criteria can be used, such as the fold change or p-value criteria with various thresholds. For instance, if the results are disappointing using the genes with fold change > 2 in at least five arrays, the statistician might decide to be more stringent and select only genes with fold change > 3. This problem may yield an additional bias. On the whole, the bias observed in this study can on one hand be considered as overestimated (because few statisticians will try all 124 classifiers), but on the other hand as underestimated (because other potential sources of bias have not been considered).

Note that an unbiased error estimate for the optimal classifier can be obtained based on validation data, if such a data set is available. Prediction tools should always be thoroughly evaluated based on fresh external validation data before their application in clinical settings [37,38]. Validation on independent data goes beyond the scope of this paper, which focuses on the developmental phase involving a CV-like procedure. However, both issues are tightly connected, since it would be a waste of time and money to start the validation phase if the results of the developmental phase are over-optimistic due to an incorrect analysis. 

As a conclusion, let us mention that the bias outlined in the present article does not only affect biomedical articles, but also potentially methodological articles, where tuning parameters or method features are often optimized based on the data sets that are subsequently used for evaluation and comparison [39].

Developing a new prediction method and evaluating it by means of comparing its performance to that of competing methods using the same data set can lead to over-optimistic conclusions in the sense that the new method's characteristics overfit the considered example data sets [39], following the mechanism illustrated in the present paper. The biased result is then the superiority of the new method rather than the prediction error itself. More research is needed to develop adequate workflows for correctly addressing this problem in biometric and bioinformatics research.

## Competing interests

The authors declare that they have no competing interests.

## Authors' contributions

ALB identified the problem, performed the analyses and drafted the manuscript. CS contributed to the concept and to the manuscript.

## Pre-publication history

The pre-publication history for this paper can be accessed here:

http://www.biomedcentral.com/1471-2288/9/85/prepub
